# Intravitreal Dexamethasone Implant in Retinal Vein Occlusion: A Pilot Study Exploring Baseline Ocular and Circulating Biomarkers

**DOI:** 10.3390/ijms27020924

**Published:** 2026-01-16

**Authors:** Carlo Gesualdo, Settimio Rossi, Fabiana Anna D’Agostino, Rosalba Casaburi, Maria Consiglia Trotta, Caterina Claudia Lepre, Marina Russo, Michele D’Amico, Francesca Simonelli

**Affiliations:** 1 Eye Clinic, Multidisciplinary Department of Medical, Surgical and Dental Sciences, University of Campania “Luigi Vanvitelli”, 80138 Naples, Italy; carlo.gesualdo@unicampania.it (C.G.); settimio.rossi@unicampania.it (S.R.); fabianaanna.dagostino@unicampania.it (F.A.D.); rosalba.casaburi.03@gmail.com (R.C.); francesca.simonelli@unicampania.it (F.S.); 2 Department of Life Sciences, Health and Health Professions, Link Campus University, 00165 Rome, Italy; 3 Department of Experimental Medicine, University of Campania “Luigi Vanvitelli”, 80138 Naples, Italy; michele.damico@unicampania.it; 4 PhD Course in National Interest in Public Administration and Innovation for Disability and Social Inclusion, Department of Mental, Physical Health and Preventive Medicine, University of Campania “Luigi Vanvitelli”, 80138 Naples, Italy; marina.russo@unicampania.it; 5 School of Pharmacology and Clinical Toxicology, University of Campania “Luigi Vanvitelli”, 80138 Naples, Italy

**Keywords:** branch retinal vein occlusion, central retinal vein occlusion, Ozurdex, ocular biomarkers, systemic biomarkers

## Abstract

This pilot study assessed the effectiveness of the intravitreal dexamethasone implant (Ozurdex) in retinal vein occlusion (RVO) patients and explored potential pre-treatment biomarkers to improve management and prognosis. Eighteen patients with branch RVO (BRVO) and twenty-four with central RVO (CRVO) receiving two intravitreal injections of Ozurdex (at baseline and between 4 and 6 months) were included. Best-corrected visual acuity (BCVA) and central retinal thickness (CRT) were recorded at baseline and after 3, 6, and 12 months. Retinal morphology was assessed using optical coherence tomography (OCT), and serum biomarkers were analyzed by ELISAs. No significant BCVA improvement was observed in RVO patients, while CRT significantly decreased from 3 to 12 months. Patients without defects of the retinal inner layers, ellipsoid zone, and external limiting membrane showed significantly higher BCVA at 6 and 12 months. Both BRVO and CRVO groups demonstrated significant BCVA improvement and CRT reduction at 6 and 12 months, with better outcomes in BRVO patients. These patients exhibited lower baseline serum levels of xanthine oxidase (XO) and thrombospondin-1 (TSP-1), which inversely correlated with BCVA at 12 months. Ozurdex was effective in real-life RVO treatment, particularly in BRVO. Serum XO and TSP-1 may serve as prognostic biomarkers for RVO.

## 1. Introduction

Retinal vein occlusion (RVO) is the second most common cause of retinal vascular disorders after diabetic retinopathy, with an overall current prevalence of 0.77% in patients aged from 30 to 89. During the late stages, RVO may cause significant visual loss, especially due to macular edema and retinal ischemia-induced neovascularization [1,2]. According to the site of occlusion, RVO can be classified as branch RVO (BRVO) or central RVO (CRVO), with BRVO showing a higher prevalence (0.64%) compared with CRVO (0.13%) [2]. Recently, the quality of RVO prognosis has markedly improved due to innovative imaging tools such as ultra-wide-field angiography (UWFA), spectral domain-optical coherence tomography (SD-OCT), and optical coherence tomography angiography (OCTA), along with several therapeutic options, such as laser therapy, intravitreal injections of Anti-Vascular Endothelial Growth Factors (VEGFs), or corticosteroid implants [3,4,5]. In this regard, several studies have shown the efficacy and safety of the slow-release intravitreal dexamethasone implant (Ozurdex) in RVO patients, which reduced visual loss over 12 months [6,7].

The exact RVO pathogenesis is still unclear, although it seems to be multifactorial, with a combination of vessel wall degeneration, venous stasis, and blood hypercoagulability [1,8,9]. Moreover, several systemic risk factors for RVO have been identified, such as hypertension, hyperhomocysteinemia (Hcys), smoking, diabetes mellitus, atherosclerosis, hypercoagulability, dyslipidemia, atrial fibrillation, and a family history of stroke [9,10,11,12,13]. Among ocular risk factors, open-angle glaucoma, hyperopia, and ocular inflammatory diseases are considered prevalent [9,10,14,15], with higher intraocular inflammatory markers correlated with RVO severity, retinal ischemia, and macular edema [16,17]. Interestingly, since high blood pressure, arteriosclerosis, and hypermetropia are often associated with BRVO pathogenesis, while intraocular pressure is more frequent in CRVO [18,19], BRVO and CRVO should be considered two different entities, each with its own prognosis and management [20].

To date, innovative retinal imaging tools, especially SD-OCT, have identified several predictive functional RVO biomarkers at the retinal level, such as disorganization of retinal inner layers (DRIL), hyperreflective foci (HRF), serous retinal detachment (SRF), ellipsoid zone (EZ) defects, and external limiting membrane (ELM) disruption [21,22,23,24]. Despite these developments, given weak evidence regarding RVO biomarkers, there is a clear need for more reliable approaches. Investigating noninvasive circulating biomarkers could represent a promising strategy for improving risk stratification and prognostic assessment in RVO. To date, although some circulating biomarkers have been associated with RVO incidence, such as circulating neutrophil extracellular trap (NET)-related markers [25,26] or serum endocan levels [27], their correlation with the RVO progression and functional results still needs to be validated. Therefore, scientific knowledge regarding systemic biomarkers with RVO prognostic relevance remains limited, highlighting the importance of further research in this field. In this regard, novel circulating biomarkers could be identified based on RVO risk factors or molecular pathways, such as inflammation, oxidative stress, retinal neovascularization, and endothelial dysfunction [18,28,29,30,31].

Therefore, the aim of the present pilot study was to explore innovative systemic biomarkers (xanthine oxidase—XO; ghrelin—GHRL; P2 purinoceptor 7—P2X7R; thrombospondin-1, TSP-1) potentially related to RVO pathogenesis and progression [32,33,34,35,36,37,38] and to correlate them with the functional results of patients with BRVO and CRVO treated for 12 months with Ozurdex intravitreal injections.

## 2. Results

At the Eye Clinic of the “Luigi Vanvitelli” University of Campania, 42 patients (26 men and 16 women) with a mean age of 72.2 ± 11 years, diagnosed with CRVO (*n* = 24) or BRVO (*n* = 18), were enrolled and followed up for a period of 12 months. A total of 42 eyes (one from each patient) were analyzed. Both RVO groups showed similar ages (CRVO: 71.5 ± 12 years, BRVO: 73.0 ± 11 years; *p* > 0.05) and sex distribution (CRVO: 29% females, BRVO: 50% females; *p* > 0.05). All patients underwent two intravitreal injections of Ozurdex at baseline and between 4 and 6 months, respectively. No rescue treatment was required during follow-up. The baseline demographic and ocular characteristics are summarized in Table 1.

### 2.1. Functional Results

**Figure 1:**
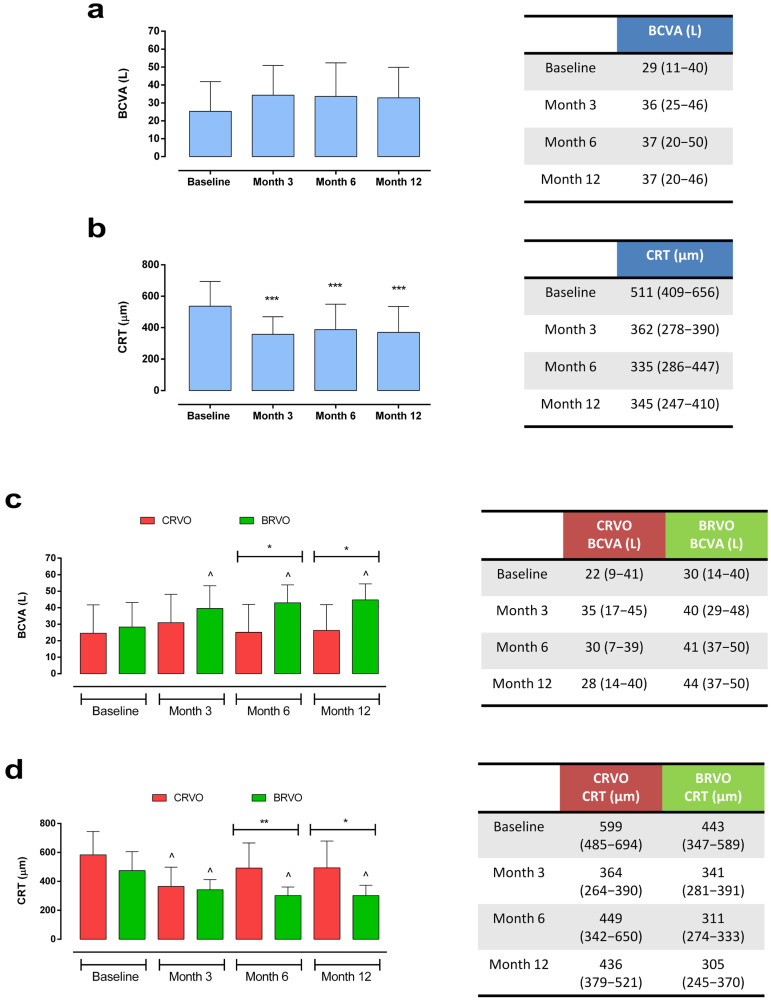
Changes in (**a**) BCVA and (**b**) CRT in RVO patients (*n* = 42) at baseline, 3, 6, and 12 months; *** *p* < 0.001 vs. baseline (Kruskal–Wallis test, followed by Dunn’s test). Changes in (**c**) BCVA and (**d**) CRT in CRVO (*n* = 24) vs. BRVO (*n* = 18) groups at baseline, 3, 6, and 12 months; * *p* < 0.05 and ** *p* < 0.01 (Mann–Whitney U test, with Holm’s correction for multiple comparisons); ^ *p* < 0.05 vs. baseline (Friedman test). Data are reported as median and interquartile range (IQR). BCVA: best-corrected visual acuity; BRVO: branch retinal vein occlusion; CRT: central retinal thickness; CRVO: central retinal vein occlusion.

Analyzing the entire RVO cohort, we observed a non-significant improvement in best-corrected visual acuity (BCVA) at 3 months [36 (25–46) L vs. 29 (11–40) L, *p* > 0.05], which remained stable until the end of the follow-up. Furthermore, a significant reduction in central retinal thickness (CRT) was observed starting at 3 months [362 (278–390) µm vs. 511 (409–656) µm, *p* < 0.001]. This remained stable at the 12-month follow-up [345 (247–410) µm, *p* < 0.001 vs. baseline] (Figure 1a,b).

**Figure 2:**
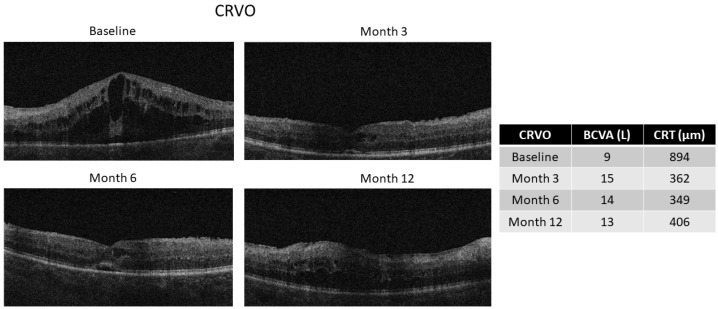
Representative OCT images of a CRVO patient receiving two Ozurdex intravitreal injections, one at baseline and one at the fifth month. Images were obtained at the baseline and after 3, 6, and 12 months. BCVA (L) and CRT (µm) values are reported for each time point. BCVA: best-corrected visual acuity; CRT: central retinal thickness; CRVO: central retinal vein occlusion; OCT: optical coherence tomography.

**Figure 3:**
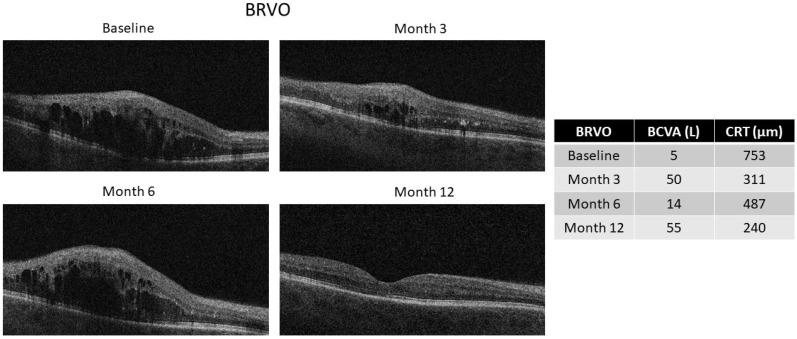
Representative OCT images of a BRVO patient receiving two Ozurdex intravitreal injections, one at baseline and one at the fifth month. Images were obtained at the baseline and after 3, 6, and 12 months. BCVA (L) and CRT (µm) values are reported for each time point. BCVA: best-corrected visual acuity; CRT: central retinal thickness; BRVO: branch retinal vein occlusion; OCT: optical coherence tomography.

Evaluating patients with BRVO vs. CRVO, we observed a significant improvement in BCVA both at 6 months [BRVO: 41 (37–50) L vs. CRVO: 30 (7–39) L, *p* < 0.05] and at 12 months [BRVO: 44 (37–50) L vs. CRVO: 28 (14–40) L, *p* < 0.05] in the BRVO group compared with the CRVO group. Similarly, we observed a statistically significant reduction in CRT at 6 months in the BRVO vs. CRVO group [BRVO: 311 (274–333) µm vs. CRVO: 449 (342–650) µm, *p* < 0.01], which was still present at the end of the 12-month follow-up [BRVO: 305 (245–370) µm vs. CRVO: 436 (379–521) µm, *p* < 0.05] (Figure 1c,d). The representative optical coherence tomography (OCT) images of patients with CRVO and BRVO during follow-up are reported in Figure 2 and Figure 3.

**Figure 4:**
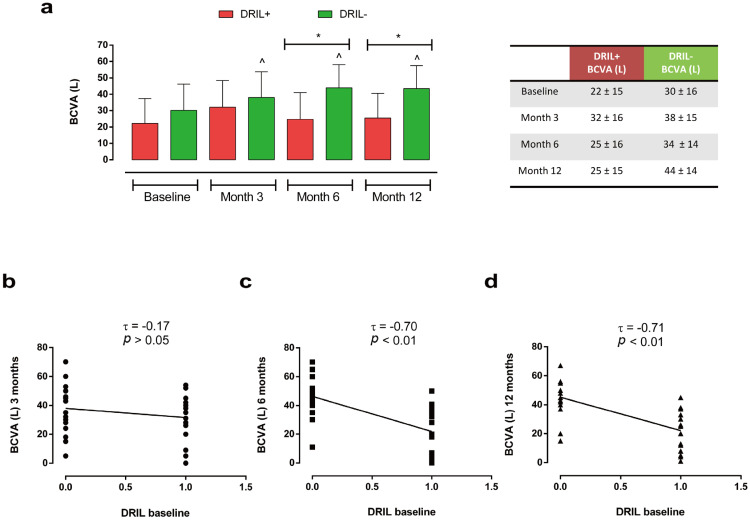
(**a**) Changes in BCVA (L) at baseline, 3, 6, and 12 months in DRIL+ (*n* = 20) vs. DRIL− (*n* = 22) groups; * *p* < 0.05; ^ *p* < 0.05 vs. baseline, same group (RM-ANOVA followed by Bonferroni’s test and Geisser–Greenhouse correction). Data are reported as mean ± standard deviation (SD). (**b**) Kendall correlation analysis of DRIL presence (value = 1) or absence (value = 0) at baseline with BCVA (L) at 3 months, (**c**) 6 months, and (**d**) 12 months. Different symbols indicate BCVA values at different follow-up times: 3 months (dot), 6 months (square), and 12 months (triangle). τ: Kendall’s Tau coefficient. BCVA: best-corrected visual acuity; DRIL: disorganization of retinal inner layers.

Regarding the BCVA variations during the follow-up based on the presence (DRIL+) or absence of DRIL (DRIL−) at baseline, we observed that only DRIL− subjects (*n* = 22) showed a significant improvement in vision at all the time points (3 months: 38 ± 15 L; 6 months: 34 ± 14 L; 12 months: 44 ± 14 L, all *p* < 0.05 vs. baseline) compared with baseline values (30 ± 16 L) (Figure 4a).

Moreover, DRIL-patients evidenced higher BCVA values both at 6 and 12 months compared with DRIL+ patients (*n* = 20) (6 months: DRIL−: 34 ± 14 L vs. DRIL+: 25 ± 16 L; 12 months: DRIL−: 44 ± 14 L vs. DRIL+: 25 ± 15 L; both *p* < 0.05), (Figure 4a), with a significant inverse correlation between the two parameters at 6 (τ = −0.70, *p* < 0.01) and 12 months (τ = −0.71, *p* < 0.01) (Figure 4b–d).

**Figure 5:**
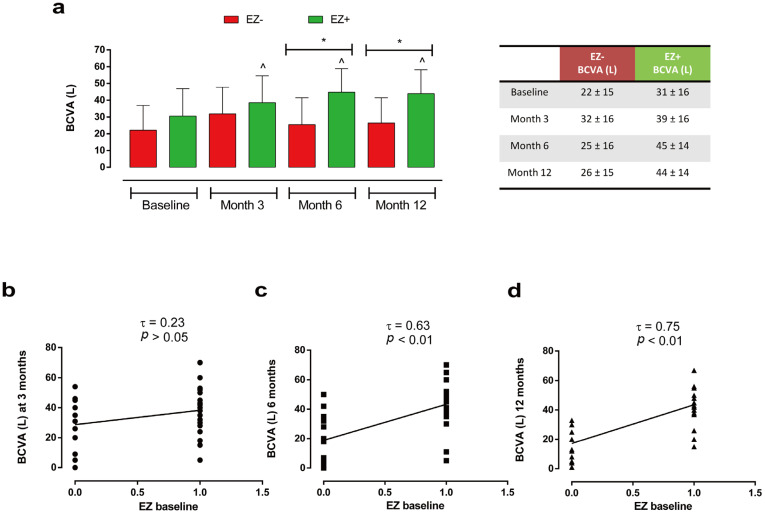
(**a**) Changes in BCVA (L) at baseline, 3, 6 and 12 months in patients with EZ defects (EZ−; *n* = 14) vs. patients with EZ integrity (EZ+; *n* = 28) groups; * *p* < 0.05, ^ *p* < 0.05 vs. baseline, same group (RM-ANOVA followed by Bonferroni’s test and Geisser–Greenhouse correction). Data are reported as mean ± standard deviation (SD). (**b**) Kendall correlation analysis of EZ integrity (value = 1) or defects (value = 0) at baseline with BCVA (L) at 3 months, (**c**) 6 months, and (**d**) 12 months. Different symbols indicate BCVA values at different follow-up times: 3 months (dot), 6 months (square), and 12 months (triangle). τ: Kendall’s Tau coefficient. BCVA: best-corrected visual acuity; ellipsoid zone (EZ).

Analyzing visual recovery during follow-up based on EZ band integrity (EZ+ group, *n* = 28) at baseline, we observed a statistically significant improvement in BCVA in the EZ+ group compared with subjects with EZ defects (EZ− group, *n* = 14) both at 6 months (EZ+: 45 ± 14 L vs. EZ−: 25 ± 16 L) (*p* < 0.05) and at 12 months (EZ+: 44 ± 14 L vs. EZ−: 26 ± 15 L) (*p* < 0.05) (Figure 5a).

Moreover, only EZ+ patients showed a significant increment in visual activity compared with their baseline BCVA (31 L ± 16 L), starting from 3 months until the end of the follow-up (3 months: 39 ± 16 L; 6 months: 45 ± 14 L; 12 months: 44 ± 14 L; all *p* < 0.05 vs. baseline) (Figure 5a). We also observed a significant direct correlation between BCVA and EZ band integrity at baseline, both at 6 (τ = 0.63, *p* < 0.01) and at 12 months (τ = 0.75, *p* < 0.01) (Figure 5b–d).

**Figure 6:**
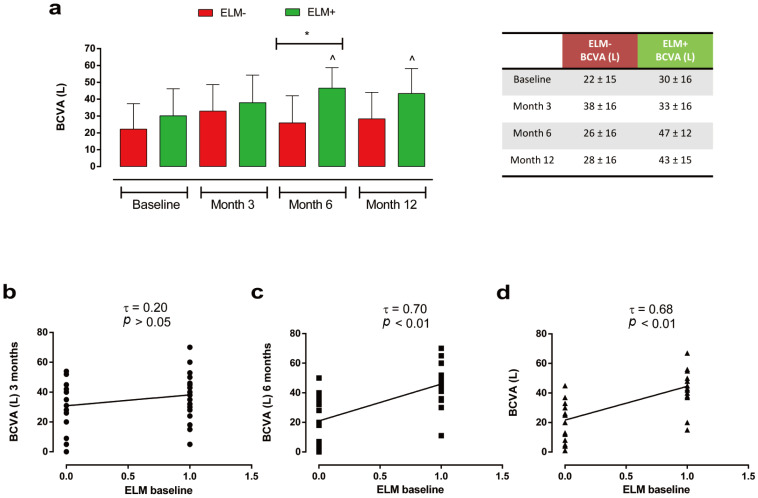
(**a**) Changes in BCVA (L) at baseline, 3, 6 and 12 months in patients with ELM disruption (ELM-; *n* = 19) vs. patients with ELM integrity (ELM+; *n* = 23) groups; * *p* < 0.05, ^ *p* < 0.05 vs. baseline, same group (RM-ANOVA followed by Bonferroni’s test and Geisser–Greenhouse correction). Data are reported as mean ± standard deviation (SD). (**b**) Kendall correlation analysis of ELM integrity (value = 1) or disruption (value = 0) at baseline with BCVA (L) at 3 months, (**c**) 6 months, and (**d**) 12 months. Different symbols indicate BCVA values at different follow-up times: 3 months (dot), 6 months (square), and 12 months (triangle). τ: Kendall’s Tau coefficient. BCVA: best-corrected visual acuity; ELM: external limiting membrane.

Similarly, we observed a significant improvement in BCVA at 6 months (in patients who had an intact ELM at baseline (ELM+ group, *n* = 23) compared with subjects with ELM disruption (ELM− group, *n* = 19) (ELM+: 47 ± 12 L vs. ELM−: 26 ± 16 L, *p* < 0.05), with a significant improvement in visual activity recorded only in ELM+ patients at 6 and 12 months (respectively, 47 ± 12 L and 43 ± 15 L; both *p* < 0.05) compared with baseline (30 L ± 16 L) (Figure 6a).

These findings were paralleled by a direct correlation between BCVA and ELM status at baseline, both at 6 (τ = 0.70, *p* < 0.01) and 12 months (τ = 0.68, *p* < 0.01) (Figure 6b–d).

Regarding Ozurdex safety, we did not observe any significant side effects except for intraocular pressure elevations observed in two patients about two months after the first intravitreal injection. These were completely reversed with the use of anti-hypertensive eye drops.

### 2.2. Serum Biomarkers

The serum biomarkers evaluated at baseline in CRVO and BRVO patients were as follows: (I) XO as a marker of oxidative stress [32,39], (II) GHRL as a modulator of retinal angiogenesis [38], (III) P2X7R as a marker for retinal neovascularization and inflammation [33,34], and TSP-1 as an inhibitor of retinal neovascularization [35,36,37]. These were compared with serum levels of healthy patients without ocular pathologies (CTRL group, *n* = 12).

**Figure 7:**
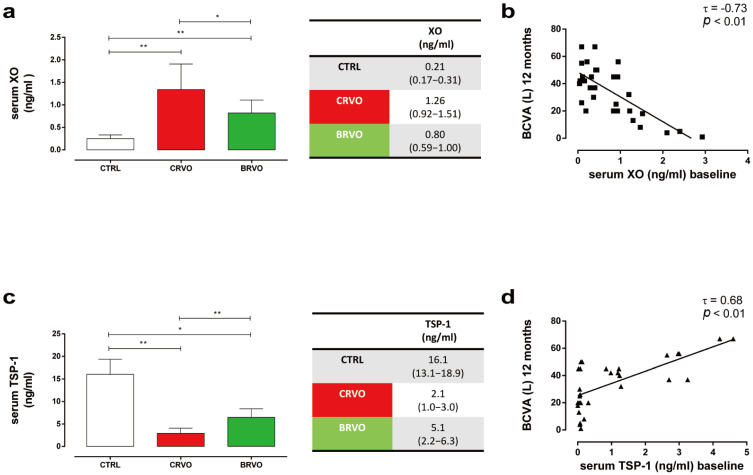
(**a**) Changes in serum XO (ng/mL) in CTRL (*n* = 12), CRVO (*n* = 24), and BRVO (*n* = 18) patients at baseline; * *p* < 0.05 and ** *p* < 0.01 (Kruskal–Wallis test, followed by Dunn’s test). Data are reported as median and interquartile range (IQR). (**b**) Kendall correlation analysis of XO serum levels (ng/mL) at baseline with BCVA (L) at 12 months. (**c**) Changes in serum TSP-1 (ng/mL) in CTRL (*n* = 12), CRVO (*n* = 24), and BRVO (*n* = 18) patients at baseline; * *p* < 0.05 and ** *p* < 0.01 (Kruskal–Wallis, followed by Dunn’s test). Data are reported as median and interquartile range (IQR). (**d**) Kendall correlation analysis of TSP-1 serum levels (ng/mL) at baseline with BCVA (L) at 12 months. τ: Kendall’s Tau coefficient; BCVA: best-corrected visual acuity; BRVO: branch retinal vein occlusion; CRVO: central retinal vein occlusion; CTRL: control; XO: xanthine oxidase; TSP-1: thrombospondin-1.

While GHRL and P2X7R serum levels did not exhibit any modulations between CTRL, CRVO, and BRVO at baseline (both *p* > 0.05) (Figure S1), serum XO was significantly up-regulated in CRVO and BRVO patients compared with the CTRL group with values of 1.26 (0.92–1.51) ng/mL and 0.80 (0.59–1.00) ng/mL, respectively, both at *p* < 0.01 vs. CTRL (Figure 7a).

Moreover, a significant serum XO decrease was observed in BRVO compared with CRVO (*p* < 0.05) (Figure 7a), with a significant inverse correlation between XO serum levels at baseline with BCVA at 12 months (τ = −0.73, *p* < 0.01) (Figure 7b).

Conversely, CRVO and BRVO patients showed a significant decrease in serum TSP-1 levels compared with CTRL [CRVO: 2.1 (1.0–3.0) ng/mL, *p* < 0.05 vs. CTRL; BRVO: 5.1 (2.2–6.3) ng/mL, *p* < 0.01 vs. CTRL] (Figure 7c). In comparison with BRVO, the CRVO group exhibited lower serum TSP-1 (*p* < 0.01) (Figure 7c), with a significantly positive association with BCVA at 12 months (τ = 0.68, *p* < 0.01) (Figure 7d).

## 3. Discussion

In recent years, increasing emphasis has been placed on characterizing new prognostic factors aiming to improve the identification of subjects with increased RVO risk and to identify poor responders to clinical treatment at baseline. This is to improve RVO outcomes through a personalized medicine approach [40].

RVO assessment has been transformed by SD-OCT, enabling both the identification and quantification of retinal functional biomarkers such as DRIL, ELM, and EZ disruption [21,40,41,42,43,44,45,46]. Moreover, several intraocular active molecules have been proposed as potential biomarkers for RVO prognosis [47]. Particularly, baseline aqueous values of soluble Intercellular Adhesion Molecule 1 (sICAM-1), soluble VEGF Receptor 1 (sVEGFR-1), and platelet-derived growth factor-isoform AA (PDGF-AA) could have a predictive role in the recurrence of RVO and consequent macular edema [48,49,50,51]. Baseline aqueous VEGF levels were higher in RVO patients unresponsive to Ranimizumab [52] and Bevacizumab [53] but not to Ozurdex [54], while baseline aqueous levels of interleukin 8 (IL-8) correlated with the insufficient efficacy of anti-VEGFs [55]. Interestingly, both baseline aqueous VEGF and IL-8 values were higher in CRVO patients compared with the BRVO population [54,56].

However, since this evidence on intraocular RVO biomarkers is still conflicting and needs to be further validated, a different approach evaluating noninvasive circulating biomarkers could be more effective for RVO risk stratification and prognosis. In this context, there is a lack of knowledge regarding systemic biomarkers with potential RVO prognostic value. Indeed, RVO incidence has been clinically associated only with serum markers of NET formation, a process related to retinal inflammation [25,26], or with endocan, a marker of endothelial cell injury [27]. Particularly, serum NET markers such as myeloperoxidase–DNA, cell-free DNA, and citrullinated histone H3 were higher in CRVO patients compared with the BRVO group [25].

Therefore, the present pilot study explored the possible involvement of new systemic biomarkers in the pathogenesis and evolution of various forms of RVO, correlating them retrospectively with the anatomical and functional outcomes obtained after 12 months of therapy with intravitreal Ozurdex injections. Starting from the third month and continuing through the end of the follow-up, the total RVO cohort evidenced a trend in BCVA improvement, although not statistically significant, in parallel with a significant CRT decrease. Moreover, our results confirmed the better functional outcomes in patients with BRVO compared with CRVO, as reported by other clinical trials [6,57]. Indeed, BCVA and CRT values were significantly improved in the BRVO subgroup from 6 months until the end of follow-up. Similarly, in line with multiple studies, at the end of the follow-up, we observed a significant correlation between the improvement in visual acuity and the presence and/or integrity at baseline of some OCT biomarkers, such as the EZ band, ELM, and DRIL [21,40,43,44,45]. Particularly, we observed that RVO patients without DRIL and with EZ band integrity were characterized by higher BCVA values starting at 6 months and continuing up to the end of follow-up. Similarly, RVO patients without ELM defects showed better BCVA outcomes at 6 months.

The exploratory aspect of our pilot study is the preliminary evaluation of circulating biomarkers that could interfere with the pathogenesis or the different evolution of RVO, eventually showing a different expression between BRVO and CRVO. Particularly, we analyzed the following serum markers: (I) XO as a marker of oxidative stress [32,39], (II) GHRL as a modulator of retinal angiogenesis [38], (III) P2X7R as a marker for retinal neovascularization and inflammation [33,34], and TSP-1 as an inhibitor of retinal neovascularization [35,36,37]. Interestingly, in this small cohort, we observed, for the first time, potential differences in the circulating levels of XO and TSP-1 in BRVO and CRVO subjects compared with healthy controls.

In this regard, XO is an enzyme present at retinal levels, particularly in the capillary endothelium cells of blood vessels [58], forming hydrogen peroxide and superoxide [32]. These interfere with the process of remethylating homocysteine into methionine, resulting in Hcys [39,59]. This is a well-known risk factor for RVO [60,61,62,63,64], causing pro-thrombotic effects, direct cytotoxic effects on retinal endothelial cells, and pro-inflammatory actions in vascular smooth muscle cells present in retinal arterioles [65,66,67,68,69,70,71]. Here, a marked increase in serum XO levels in CRVO compared with BRVO patients is reported for the first time, in line with a previous study by Lahiri et al. reporting higher Hcys in the CRVO compared with the BRVO group [60]. Furthermore, our results showed that serum XO levels in the RVO population were inversely correlated with BCVA at the end of follow-up. This trend may suggest a potential association between oxidative stress and poorer visual prognosis in CRVO patients. Indeed, other oxidative stress markers such as malondialdehyde, 8-hydroxy-2-deoxyguanosine, and hydrogen peroxide were previously correlated with RVO [72,73]. However, no differences between BRVO and CRVO patients, nor any correlations with functional outcomes, have been evidenced for these molecules [72,73].

Similarly, we also found a significant dysregulation of TSP-1 serum levels in our pilot study. TSP-1 is a matricellular protein mainly produced by retinal glial cells and is able to inhibit retinal angiogenesis, characterizing RVO [74,75,76,77,78,79]. Indeed, TSP-1 silencing in mice was associated with an increased retinal vascular density and with an insufficient recovery of retinal vessel damage during oxygen-induced ischemic retinopathy [80,81]. To date, serum TSP-1 levels have not yet been associated with retinal angiogenesis in clinical settings; however, elevated TSP-1 circulating levels have been found in patients with endothelial dysfunction, although without retinopathy [82,83]. Here we found lower serum TSP-1 levels in patients with CRVO compared with those with BRVO at baseline, paralleling the worse BCVA outcomes observed in CRVO. These findings raise the possibility that reduced TSP-1 serum levels may be linked to a higher susceptibility to ischemia-driven retinal changes in CRVO patients. In conclusion, our evidence confirmed the efficacy of intravitreal Ozurdex in the RVO population, with better outcomes in BRVO patients compared with CRVO. Moreover, in our clinical setting, XO and TSP-1 appear to be promising serum biomarkers that may hold potential prognostic value in RVO management. However, our results have some limitations. We hypothesized that the serum variation in the markers was primarily due to RVO itself rather than other systemic conditions (such as controlled diabetes and/or hypertension), since both age and general health status were similar among control subjects and patients with different forms of RVO. It is possible that the dysregulation of these markers could be caused by other systemic conditions related to RVO development or linked to the different sex distribution between BRVO and CRVO patients. Notably, our sample size is relatively small, leading to potential bias and limiting the reliability and generalizability of the findings. Therefore, further studies with a larger sample size are needed to confirm the association between RVO prognosis and new ocular and systemic biomarkers.

## 4. Materials and Methods

### 4.1. Study Design

This is a pilot study in which 42 patients (42 eyes) diagnosed with RVO were enrolled from June 2024 to December 2024 at the Eye Clinic of the University of Campania “Luigi Vanvitelli” in Naples, Italy, where they were followed and treated for 12 months. The study was conducted according to the principles outlined in the Declaration of Helsinki and received approval from the Board of Reviewers of the University of Campania “Luigi Vanvitelli” (Prot. 0013698/I, 9 May 2024). All enrolled patients provided informed consent to participate in the study. The following systemic exclusion criteria were adopted: uncontrolled diabetes and/or hypertension; use of immunosuppressive drugs and/or non-steroidal/steroidal anti-inflammatory drugs or lipid-lowering agents; infections, cardiovascular or cerebrovascular events in the last 6 months; and nephropathy and/or ketoacidosis. Moreover, we also considered the following ocular exclusion criteria: previous treatments with a slow-release intravitreal insert of dexamethasone or anti-VEGFs in the last 12 months; concomitant ocular diseases such as age-related macular degeneration, hereditary retinal dystrophies, diabetic retinopathy, and uveitis; and vitrectomy performed within the last 6 months.

We divided the entire cohort into two groups based on the type of retinal vein occlusion: Group 1: subjects with BRVO (*n* = 18) and Group 2: subjects with CRVO (*n* = 24). All participants received two Ozurdex injections (Abbvie S.r.l., Rome, Italy) for post-occlusive macular edema at baseline and between 5 and 6 months, respectively.

All patients underwent a comprehensive ophthalmologic evaluation, which included the assessment of BCVA using the Early Treatment Diabetic Retinopathy Study (ETDRS) chart at 2 m; anterior segment biomicroscopy and binocular indirect ophthalmoscopy; and SD-OCT, OCTA, and Goldmann applanation tonometry. SD-OCT and OCTA imaging were performed with the Zeiss Engineering Cirrus 6000 (Oberkochen, Germany), using the 512 × 128 Macular Cube protocol. CRT was measured at the center of the fovea as the distance between the retinal surface and the retinal pigment epithelium. The presence and integrity of the following OCT biomarkers were also assessed at baseline: DRIL, EZ, and ELM. Baseline and follow-up images were independently graded by two investigators (F.D.A., R.C.) and verified by a senior colleague (C.G.).

For the evaluation of pre-treatment serum biomarkers, 12 healthy patients without ocular pathologies were enrolled and used as controls (CTRL group; *n* = 12). These were matched for age and sex distribution with the RVO population. All of them provided informed consent to participate in the study. Their demographics are described in Table 2.

### 4.2. Study Endpoints

The primary endpoint was the analysis of changes in BCVA and CRT at 3, 6, and 12 months in the entire cohort. Subsequently, any differences in terms of both visual recovery (BCVA) and anatomical changes (CRT) between the two patient groups (BRVO vs. CRVO) were studied.

Furthermore, all correlations between the functional results obtained at the end of follow-up, with the presence of DRIL and/or with the EZ band and ELM status at baseline, were retrospectively analyzed.

Finally, the correlation between visual results and new serum biomarkers related to the risk of RVO, such as XO, ET-1, P2X7R, and TSP-1 (all assessed at baseline), was retrospectively evaluated.

### 4.3. Serum Collection and Enzyme-Linked Immunosorbent Assays (ELISAs)

Fasting venous blood samples were collected from CTRL (*n* = 12), BRVO (*n* = 12), and CRVO (*n* = 21) patients at baseline using sterile and dry vacutainer tubes. Within 2 h of whole blood collection, they were incubated at 20 °C for 30 min and then centrifuged at 4 °C for 15 min at 3000 rpm. Sera were aliquoted and stored at −80 °C for the subsequent analysis. Serum levels of GHRL (EH0355 Human Ghrelin ELISAS Kit, FineTest—Wuhan, China), P2X7R (CSB-EL017325HU, Houston, TX, USA), TSP-1 (MBS701627, MyBioSource; San Diego, CA, USA), and XO (EH1036, FineTest; Palm Coast, FL, USA) were assessed by commercial ELISA kits, according to the manufacturer’s protocols.

### 4.4. Statistical Analysis

Levene’s test and the Shapiro–Wilk test were used to assess data homogeneity and distribution. For non-parametric variables based on repeated observations, within-group temporal differences were analyzed using the Friedman test, whereas between-group differences at each time point were assessed using the Mann–Whitney U test, with Holm’s correction applied for multiple comparisons. Parametric variables based on repeated observations were analyzed using repeated-measures analysis of variance (RM-ANOVA) followed by Bonferroni’s multiple comparison test. Mauchly’s test was performed to assess sphericity, applying the appropriate correction (Huynh-Feldt or Greenhouse–Geisser) if necessary. RM-ANOVA data were reported as mean ± standard deviation (SD), while skewed data were presented as median (interquartile range—IQR). Kendall correlation analysis was used to assess the association between two variables, reported as Kendall’s Tau coefficient (τ). A *p*-value of *p* < 0.05 was considered statistically significant.

## Figures and Tables

**Table 1:** Baseline demographic and ocular characteristics.

RVO Patients Enrolled, *n*	42
Males; *n* (%)	26 (62%)
Females; *n* (%)	16 (38%)
Age, years; (mean ± SD)	72.2 ± 11
BVCA, L; median (IQR)	29 (11–40)
CRT, μm; median (IQR)	511 (409–656)
DRIL; *n* (%)	20 (48%)
EZ defects; *n* (%)	14 (33%)
ELM disruption; *n* (%)	19 (45%)
Controlled diabetes *; *n* (%)	16 (38%)
Controlled hypertension ^; *n* (%)	29 (69%)
CRVO; *n* (%)	24 (57%)
CRVO age, years; (mean ± SD)	71.5 ± 12
CRVO males/females; *n*	17/7
BRVO; *n* (%)	18 (43%)
BRVO age, years; (mean ± SD)	73.0 ± 11
BRVO males/females; *n*	9/9

* Controlled diabetes: glycated hemoglobin < 7%; blood sugar levels between 80 and 130 mg/dL before a meal and <180 mg/dL after a meal. ^ Controlled hypertension: systolic blood pressure < 140 mmHg and diastolic blood pressure < 90 mmHg. BCVA: best-corrected visual acuity; BRVO: branch retinal vein occlusion; CRT: central retinal thickness; CRVO: central retinal vein occlusion; DRIL: disorganization of retinal inner layers; EZ: ellipsoid zone; ELM: external limiting membrane; IQR: interquartile range; *n*: number; SD: standard deviation; %: percentage.

**Table 2:** Baseline demographics of patients without ocular pathologies.

Control Patients, *n*	12
Males; *n* (%)	7 (58%)
Females; *n* (%)	5 (42%)
Age, years; (Mean ± SD)	70.2 ± 10

*n*: number; SD: standard deviation; %: percentage.

## Data Availability

All data relevant to the study are included within the article and its Supplementary Materials.

## Supplementary Materials

The following supporting information can be downloaded at: https://www.mdpi.com/article/10.3390/ijms27020924/s1.

## Abbreviations

The following abbreviations are used in this manuscript:

BCVA	Best-corrected visual acuity
BRVO	Branch retinal vein occlusion
CRT	Central retinal thickness
CRVO	Central retinal vein occlusion
CTRL	Control
DRIL	Disorganization of retinal inner layers
ELM	External limiting membrane
EZ	Ellipsoid zone
ETDRS	Early Treatment Diabetic Retinopathy Study
GHRL	Ghrelin-1
HRF	Hyperreflective Foci
Hcys	Hyperhomocysteinemia
IL-8	Interleukin 8
NET	Neutrophil extracellular trap
OCTA	Optical coherence tomography angiography
P2X7R	P2 Purinoreceptor 7
PDGF-AA	Platelet-derived growth factor-isoform AA
RVO	Retinal vein occlusion
SD-OCT	Spectral domain-optical coherence tomography
sICAM-1	Circulating Intercellular Adhesion Molecule 1
sVEGFR-1	Soluble Vascular Endothelial Growth Factor Receptor type 1
SRF	Serous retinal detachment
TSP-1	Thrombospondin-1
UWFA	Ultra-wide-field angiography
VEGF(s)	Vascular Endothelial Growth Factor(s)
XO	Xanthine oxidase
