# Supplementary material for: Intravitreal Dexamethasone Implant in Retinal Vein Occlusion: A Pilot Study Exploring Baseline Ocular and Circulating Biomarkers
Source: Int J Mol Sci. 2026 Jan 16;27(2):924. doi: 10.3390/ijms27020924 (PMC12842590; doi:10.3390/ijms27020924)
Supplement: Supplementary file 1 [file ijms-27-00924-s001.zip › ijms-3981264-supplementary.pdf]

SUPPLEMENTARY MATERIALS

Supplementary Figures

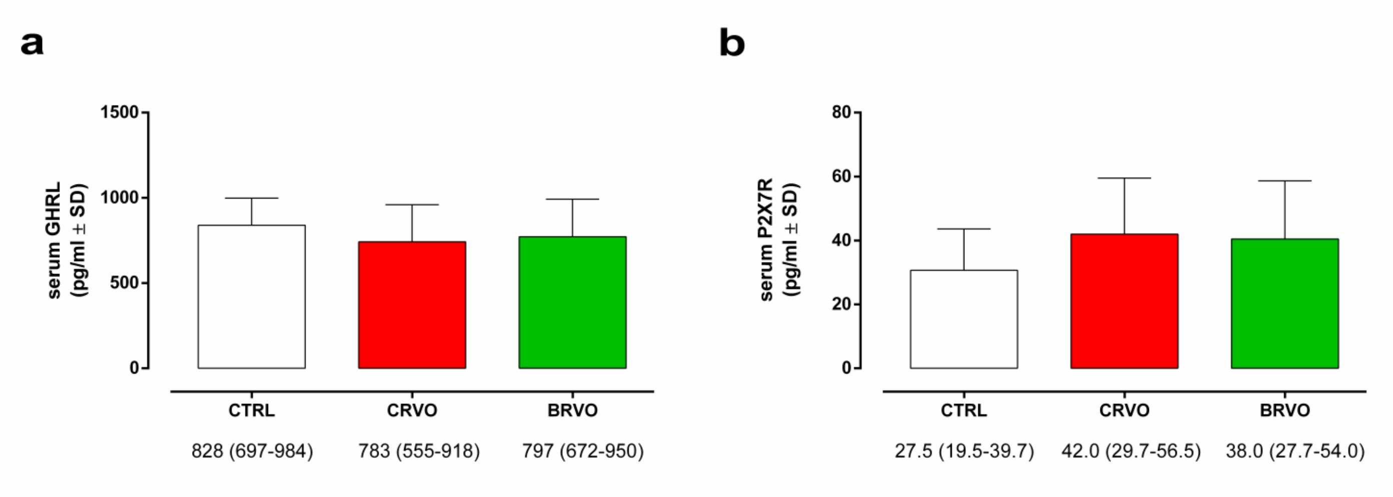

Figure S1: Serum levels of **a)** GHRL (pg/ml) and **b)** P2X7R (pg/ml) in CTRL (n = 12), CRVO (n = 24) and BRVO (n = 18) patients at baseline. Data were analyzed by Friedman's test, followed by Dunn's test. Data are reported as median and interquartile range (IQR). BRVO: branch retinal vein occlusion; CTRL: control; CRVO: central retinal vein occlusion; GHRL: ghrelin; P2X7R: P2 purinoceptor 7.
